# Short-Term Physical Inactivity Induces Endothelial Dysfunction

**DOI:** 10.3389/fphys.2021.659834

**Published:** 2021-04-09

**Authors:** Kelly A. Bowden Davies, Juliette A. Norman, Andrew Thompson, Katie L. Mitchell, Joanne A. Harrold, Jason C. G. Halford, John P. H. Wilding, Graham J. Kemp, Daniel J. Cuthbertson, Victoria S. Sprung

**Affiliations:** ^1^Department of Sport and Exercise Sciences, Manchester Metropolitan University, Manchester, United Kingdom; ^2^Institute of Life Course and Medical Sciences, University of Liverpool, Liverpool, United Kingdom; ^3^Obesity and Endocrinology Research Group, Clinical Sciences Centre, Liverpool University Hospitals NHS Foundation Trust, Liverpool, United Kingdom; ^4^Institute of Systems, Molecular and Integrative Biology, University of Liverpool, Liverpool, United Kingdom; ^5^Institute of Public Health, University of Liverpool, Liverpool, United Kingdom; ^6^School of Psychology, University of Leeds, Leeds, United Kingdom; ^7^Liverpool Magnetic Resonance Imaging Centre, University of Liverpool, Liverpool, United Kingdom; ^8^Research Institute for Sport and Exercise Sciences, Liverpool John Moores University, Liverpool, United Kingdom

**Keywords:** cardiorespiratory fitness, endothelial function, insulin resistance, liver fat, physical activity, sedentary behavior

## Abstract

**Objective:**

This study examined the effects of a short-term reduction in physical activity, and subsequent resumption, on metabolic profiles, body composition and cardiovascular (endothelial) function.

**Design:**

Twenty-eight habitually active (≥10,000 steps/day) participants (18 female, 10 male; age 32 ± 11 years; BMI 24.3 ± 2.5 kg/m^2^) were assessed at baseline, following 14 days of step-reduction and 14 days after resuming habitual activity.

**Methods:**

Physical activity was monitored throughout (SenseWear Armband). Endothelial function (flow mediated dilation; FMD), cardiorespiratory fitness ($\dot{\text{V}}\,{\text{O}}_{2}$ peak) and body composition including liver fat (dual-energy x-ray absorptiometry and magnetic resonance spectroscopy) were determined at each assessment. Statistical analysis was performed using one-way within subject’s ANOVA; data presented as mean (95% CI).

**Results:**

Participants decreased their step count from baseline by 10,111 steps/day (8949, 11,274; *P* < 0.001), increasing sedentary time by 103 min/day (29, 177; *P* < 0.001). Following 14 days of step-reduction, endothelial function was reduced by a 1.8% (0.4, 3.3; *P* = 0.01) decrease in FMD. Following resumption of habitual activity, FMD increased by 1.4%, comparable to the baseline level 0.4% (–1.8, 2.6; *P* = 1.00). Total body fat, waist circumference, liver fat, whole body insulin sensitivity and cardiorespiratory fitness were all adversely affected by 14 days step-reduction (*P* < 0.05) but returned to baseline levels following resumption of activity.

**Conclusion:**

This data shows for the first time that whilst a decline in endothelial function is observed following short-term physical inactivity, this is reversed on resumption of habitual activity. The findings highlight the need for public health interventions that focus on minimizing time spent in sedentary behavior.

## Introduction

Epidemiological evidence demonstrates that *physical inactivity* (defined as physical activity too low to meet physical activity guidelines) and *sedentary behavior* (defined as any waking behavior characterized by an energy expenditure < 1.5 metabolic equivalents (METS), while in a sitting, reclining or lying posture) play a role in the development of obesity, insulin resistance, type 2 diabetes and cardiovascular disease (CVD) (Lee et al., 2012; Henson et al., 2013; Smith et al., 2016; Tremblay et al., 2017). However, such evidence provides no mechanistic insight into the underlying pathophysiological changes associated with these behavior profiles. Whilst we understand that a physically active lifestyle is protective against CVD (Paffenbarger et al., 2001), the specific cardiovascular consequences of physical inactivity and/or increased sedentary time have not been comprehensively evaluated.

Mechanistic studies of reduced physical activity in humans have used extreme experimental models including prolonged bed rest (Alibegovic et al., 2010), limb immobilization (Abadi et al., 2009), and complete cessation of exercise in exercise-trained volunteers (King et al., 1988). These models are not physiologically representative of habitual activity levels in free-living individuals and must therefore be interpreted with caution. More recently, an alternative experimental model has been adopted in which physically active (∼10,000 steps/day) volunteers, who do not participate in regular exercise, transition to an inactive lifestyle (reducing to ∼1500 steps/day) for brief periods (∼14 days) (reviewed in Bowden Davies et al., 2019). This approach reflects societal changes in physical activity levels (i.e., reduced physical activity and more sedentary time) promoted by occupational changes and technological advances (Church et al., 2011; Althoff et al., 2017). Step-reduction models have clearly demonstrated that reduced physical activity is associated with detrimental physiological changes, including reduced cardiorespiratory fitness, accumulation of central fat, loss of skeletal muscle mass with associated anabolic resistance, and reduced peripheral insulin sensitivity (Olsen et al., 2008; Krogh-Madsen et al., 2010; Knudsen et al., 2012; Breen et al., 2013; McGlory et al., 2017; Bowden Davies et al., 2018). Endothelial function, an early prognostic and reversible marker of CVD, has been studied when transitioning individuals to ∼5000 steps/day for 5 days (Boyle et al., 2013; Restaino et al., 2016; Teixeira et al., 2017). Endothelial function was reduced but any changes on resumption of usual physical activity has not been studied. The endothelium plays a pivotal role in vascular homeostasis (Furchgott and Zawadzki, 1980), and brachial artery flow-mediated dilation (FMD) has been shown to be predictive of future CVD risk (Green et al., 2011).

We evaluated endothelial function in a specific subset of individuals from our previously published step-reduction cohort (Bowden Davies et al., 2018), and present for the first time changes in endothelial function following a 14 day reduction to ∼1,500 steps/day with subsequent resumption to usual activity. The primary aim of this study was to investigate the cardiovascular-specific consequences of 14-days decreased physical activity with increased sedentary behavior in free-living individuals, hypothesizing that endothelial function would decline but return to baseline after a following 14-day activity resumption. Other markers of physical health are reported to evaluate the context of changes accompanying alterations in endothelial function.

## Materials and Methods

### Study Design

A mean daily step count ≥10,000 was required for participants to be eligible. Physical activity screening was blinded and consisted of monitoring from midnight to midnight on 4 consecutive days, including 1 weekend day (accelerometer details below). If eligible, participants underwent their initial assessment visits before being instructed to reduce their activity to ∼1500 steps for 14 days after which the assessments were repeated. The reduction to 1500 steps/day was based on methodology from previously reviewed studies (Bowden Davies et al., 2019) and unpublished data from our group that has assessed the habitual activity of patients with type 2 diabetes. Habitual activity was then resumed for a further 14 days before participants underwent their final assessment visits. There were two assessment visits at each time point: (1) at Liverpool University Hospitals (Aintree site) for anthropometry, flow mediated dilation (FMD), fasting biochemistry, oral glucose tolerance test (OGTT) and $\dot{\text{V}}\,{\text{O}}_{2}$ peak; and (2) at the University of Liverpool for dual-energy x-ray absorptiometry (DXA) and proton magnetic resonance spectroscopy (^1^H-MRS).

The study took place between December 2014 and August 2017, and 28 participants successfully completed the intervention with all measures available. Dietary records were taken in the 4 days preceding an assessment visit; participants were instructed to maintain their usual dietary habits throughout the study.

### Participants

Habitually active participants with no history/current engagement in regular structured exercise (>2 h/week) or highly physical employment (determined by questionnaire) were recruited from local advertisements across hospital departments and university campuses. Exclusions included: cardiovascular, respiratory, kidney, liver, and/or endocrine complications, smokers and those consuming > 14 units/week of alcohol consumption. The study conformed to the *Declaration of Helsinki* and was approved North West – Liverpool Central ethics committee (14/NW/1147 and 14/NW/1145). All participants were informed of the methods verbally and in writing before providing written informed consent prior to any assessments. Before each visit, participants were required to fast overnight for >8 h, abstain from alcohol and caffeine for 24 h and from any formal exercise for 48 h.

### Experimental Measures

#### Anthropometric Measurements

Weight, height (to the nearest 0.5 cm) using a stadiometer (Model 220, Seca, Germany), and waist (umbilicus) and hip (greater trochanter) circumferences, were measured in duplicate (retested if not within 5 mm). Participants then rested for 5 min before BP was determined from an average of three measures (Dinamap, G&E Medical, United States).

#### Biochemical Measurements

Blood samples were collected and analyzed using the Olympus AU2700 analyzer (Beckman Coulter, High Wycombe, United Kingdom) with standard proprietary reagents as follows: glucose with hexokinase, total cholesterol and HDL-cholesterol with cholesterol esterase/oxidase and triacylglycerol with glycerol kinase. LDL-cholesterol was calculated according to the Friedewald formula. Insulin was measured using radioimmunoassay (Invitrogen, Paisley, United Kingdom). HOMA-IR was calculated using fasting glucose and insulin concentrations (Matthews et al., 1985). The intra- and inter-assay coefficient of variation for all blood parameters was ≤5%.

#### Oral Glucose Tolerance Test

After fasting blood samples were collected, a 75 g glucose solution was consumed within 5 min and post-ingestion blood samples were drawn at 30–, 60-, 90-, and 120-min. Glucose, insulin and NEFA responses were calculated as AUC. Matsuda index was calculated to estimate whole-body insulin sensitivity (Matsuda and DeFronzo, 1999).

#### Cardiorespiratory Fitness

A $\dot{\text{V}}\,{\text{O}}_{2}$ peak cardiopulmonary exercise test (CPET) was performed on a treadmill (Model 77OCE, RAM Medisoft Group, Manchester, United Kingdom) in a temperature-controlled room. The CPET provided breath-by-breath monitoring and analysis of expiratory gases and ventilation as well as continuous electrocardiographic monitoring (Love Medical Cardiopulmonary Diagnostics, Manchester, United Kingdom). The modified Bruce protocol was employed: after an initial 2 min warm up at 2.2 km/h on a flat gradient, stepwise increments in speed and gradient were made each min. $\dot{\text{V}}\,{\text{O}}_{2}$ peak was determined by any two of: respiratory exchange ratio > 1.15; heart rate > 90% predicted maximum; plateau in $\dot{\text{V}}\,{\text{O}}_{2}$; or exhaustion. $\dot{\text{V}}\,{\text{O}}_{2}$ peak data were expressed in three ways: absolute (l/min), relative to total body mass (ml min^–1^ kg^–1^), and relative to lean body mass (ml min^–1^ kg^–1^; termed ‘$\dot{\text{V}}\,{\text{O}}_{2}$ peak lean’).

#### Physical Activity Monitoring

Physical activity was tracked throughout using a SenseWear mini armband (BodyMedia, Pittsburgh, PA, United States). Instructions were that the armband was to be worn at all possible times, and the inclusion criterion was >90% wear time, which was monitored using SenseWear Professional software (version 8.0). Data collected from the armband included: daily average step count; total energy expenditure; active energy expenditure; and time spent in domains of physical activity, including sleep, lying, sedentary (<1.5 METS), light (1.5–3 METS), moderate (3–6 METS), vigorous (6–9 METS), and very vigorous (>9 METS).

#### Dietary Analysis

Total energy consumption, carbohydrate, protein, and fat content were determined from dietary records by a registered nutritionist (KLM) using Nutritics (Nutrition Analysis Software for Professionals^1^; accessed 17/07/2017).

#### Dual-Energy X-Ray Absorptiometry

Whole-body scans were performed in line with manufacturer’s guidelines using Lunar iDXA (GE Healthcare, Amersham, United Kingdom), and were analyzed using the instrument’s software (version 13.60.033) to determine body fat, lean body mass and bone mineral density. Each scan session was preceded by a calibration routine, using multiple quality-control phantoms that simulate soft tissue and bone.

#### ^1^H-MRS

Liver and skeletal muscle fat were determined using a 1.5 T Siemens Symphony MRI scanner (Siemens Medical Solutions, Erlangen, Germany) as previously described (Cuthbertson et al., 2012; Jones et al., 2012; Cuthbertson et al., 2016). In brief, intrahepatocellular lipid (IHCL; liver fat) is expressed as the percentage of CH_2_ lipid signal amplitude relative to water signal amplitude and intramyocellular lipid (IMCL; skeletal muscle lipid) is expressed as CH_2_ lipid amplitude relative to total creatine amplitude.

#### Brachial Artery Flow Mediated Dilation (FMD)

Endothelial function was assessed by measuring FMD in response to a 5 min ischemic stimulus, induced by inflation of a forearm cuff placed immediately distal to the olecranon process, as previously described (Sprung et al., 2014) and in line with expert recommendations (Thijssen et al., 2019). Briefly, baseline images were recorded for 1 min prior to forearm cuff inflation (∼220 mmHg) for 5 min. Artery diameter and blood flow velocity recordings resumed 30 s prior to cuff deflation and continued for 3 min thereafter. Peak brachial artery diameter and blood flow velocity, and the time taken to reach these peaks following cuff release were recorded. Post-test analysis of brachial artery diameter was undertaken using custom-designed automated edge-detection and wall-tracking software.

#### Sample Size Calculation

The primary outcome variable was change in FMD. Previous studies have examined vascular function following 5 days of physical inactivity (Boyle et al., 2013) and detected differences in popliteal but not brachial artery FMD. However, given the increased longevity of our intervention (14 days as opposed to 5), and more profound reduction in physical activity (reduction to from >10,000 steps to 1,500 steps per day rather than 5,000), we would expect a detrimental effect on FMD systemically (in upper limbs as well as lower). Using a paired *t*-test with a 0.05 two-sided significance level, a sample size of 26 would have 90% power to detect a difference in means of 1.2%, assuming that the standard deviation in change score for both groups is 1.5 (G^∗^Power 3.1.5).

#### Statistical Analysis

Statistical analysis was performed using SPSS for Windows (Version 24.0, SPSS, Chicago, IL, United States). All continuous data were explored for normality using visual inspection of frequency distribution, and logarithmically transformed where appropriate. Data were back transformed to original units for reporting. The outcome variables (e.g., FMD) were analyzed using a repeated measures ANOVA. Statistically significant main effects were further explored through pairwise comparisons between timepoints with the Bonferroni approach to multiple testing. All FMD data were analyzed, and are presented, following allometric scaling which uses baseline artery diameter measured prior to the introduction of hyperemia in each test as a covariate; this approach is more accurate for scaling changes in artery diameter than simple percentage change (Atkinson and Batterham, 2013; Atkinson et al., 2013). Distribution data are presented as mean ± SD and outcomes of ANOVA as mean (95% CI). The alpha level of statistical significance was set at *P* < 0.05.

## Results

### Baseline Characteristics

Twenty-eight habitually active participants (18 female, 10 male; age 32 ± 11 years; BMI 24 ± 2.4 kg/m^2^) successfully completed the intervention with all measures; baseline characteristics are shown in Table 1.

**TABLE 1:** Participant characteristics at baseline.

Characteristic	Mean ± SD
**Clinical**	
Age (years)	32 ± 11
Body mass (kg)	71.5 ± 9.7
BMI (kg m^–2^)	24.0 ± 2.4
Waist circumference (cm)	86 ± 8
Hip circumference (cm)	98 ± 6
Waist: hip ratio	0.9 ± 0.1
SBP (mmHg)	119 ± 14
DBP (mmHg)	73 ± 8
**Glucose regulation**	
Whole-body IS (Matsuda index)^*a*^	3.4 ± 1.7
HOMA-IR	3.8 ± 1.9
**Lipid profile**	
Cholesterol (mmol/l)	4.9 ± 0.9
Triglyceride (mmol/l)^*a*^	0.9 ± 0.6
HDL-cholesterol (mmol/l)	1.9 ± 0.5
LDL-cholesterol (mmol/l)	2.6 ± 0.9
Cholesterol:HDL-cholesterol ratio	2.7 ± 0.8
**Body composition**	
Total body fat (%)	29.6 ± 8.7
Android fat (%)	30.0 ± 10.3
Gynoid fat (%)	33.1 ± 10.7
Total lean mass (kg)	48.2 ± 9.2
Leg lean mass (kg)	16.9 ± 3.3
Arm lean mass (kg)	5.4 ± 1.7
IHCL (%)^*a*^	0.6 ± 0.6
IMCL (%)	7.2 ± 4.1
**Cardiorespiratory fitness and physical activity**	
$\dot{\text{V}}\,{\text{O}}_{2}$ (l min^–1^)	2.6 ± 0.5
$\dot{\text{V}}\,{\text{O}}_{2}$ peak (ml min^–1^ kg^–1^)	36.1 ± 6.1
$\dot{\text{V}}\,{\text{O}}_{2}$ peak lean (ml min^–1^ kg^–1^)	53.3 ± 5.0
Daily total energy expenditure (kJ day ^–1^)^*a*^	11739 ± 2912
Daily steps (steps)^*a*^	12624 ± 2064
Daily total sedentary time (min)	998 ± 112
Daily waking sedentary time (min)	597 ± 94
Daily light activity (min)	224 ± 67
Daily moderate-vigorous activity (min)^*a*^	165 ± 86

*^*a*^Variables analyzed following logarithmic transformation. IR, insulin resistance; IS, insulin sensitivity; IHCL, intrahepatocellular lipid (liver fat); IMCL, intramyocellular lipid (skeletal muscle lipid).*

### Changes in Pattern of Physical Activity

**FIGURE 1:**
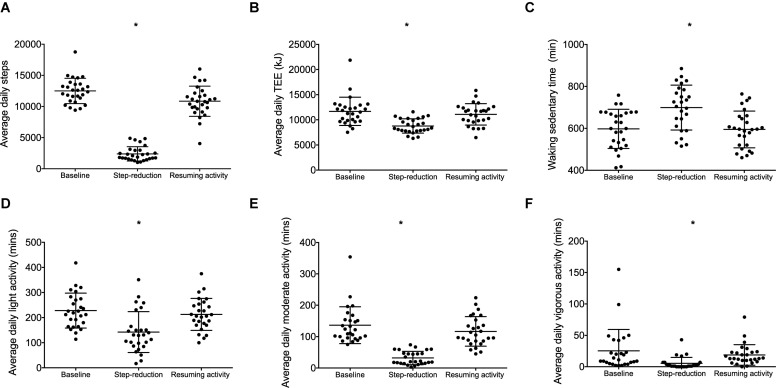
Physical activity data for individuals at baseline, following step-reduction and resuming activity, including daily average step count **(A)**, TEE **(B)**, daily waking sedentary time (<1.5 METS) **(C)**, daily light activity (1.5–3 METS) **(D)**, daily moderate activity (3–6 METS) **(E)**, and daily vigorous activity (>6 METS) **(F)**. Data are presented as mean ± SD; *P < 0.05 main effect of time.

Daily step count decreased from baseline during step-reduction by 10,111 steps/day (8949, 11,274; Figure 1A) increasing waking sedentary time by 103 min/day (29, 177; Figure 1C). Full physical activity data is shown in Figure 1; all intensities of activity > 3 METS decreased significantly during step-reduction. There were no significant differences in any physical activity measures between baseline and following resumption of activity, showing that habitual physical activity levels were successfully restored and sedentary time comparable to baseline (*P* > 0.05).

### Dietary Analysis

Total energy consumption did not change throughout the intervention (*P* > 0.05). Specifically, macronutrient percentages were 56 ± 15% carbohydrate, 24 ± 10% protein, and 20 ± 9% fat. Total energy consumption (KJ), carbohydrates (g), protein (g), and fat (g) are provided in Supplementary Table 1.

### Clinical Characteristics

Body mass, BMI and waist: hip ratio did not change during the intervention (*P* = 0.240, *P* = 0.263, and *P* = 0.491, respectively) whereas waist circumference increased by 0.8 cm (0.3, 1.3; *P* = 0.002) after step-reduction. Systolic BP and diastolic BP remained unchanged during the intervention (*P* = 0.904 and *P* = 0.914, respectively).

### Brachial Artery Flow Mediated Dilation (FMD)

**FIGURE 2:**
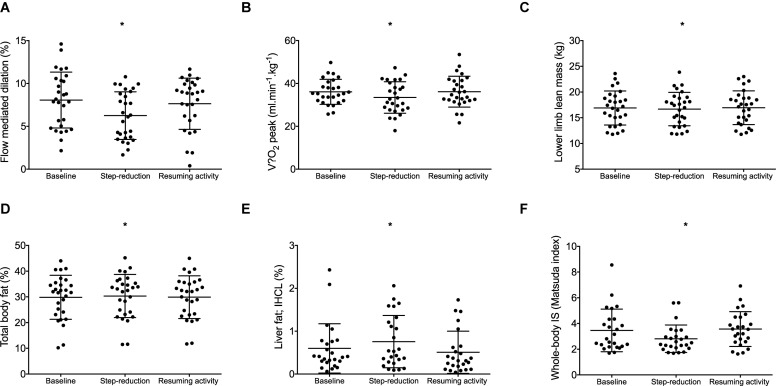
Data for individuals at baseline, following step- reduction and resumption of activity. Flow mediated dilation; FMD **(A)**, $\dot{\text{V}}\,{\text{O}}_{2}$ peak **(B)**, lower limb lean mass **(C)**, total body fat **(D)**, liver fat; IHCL **(E)**, and whole-body IS **(F)**. Data are presented as mean ± SD; *P < 0.05 main effect of time.

A significant effect of physical activity was identified (*P* = 0.04; Figure 2A). *Post hoc* comparisons revealed that FMD decreased by 1.8% (0.4, 3.3; *P* = 0.01) following 14 days of step-reduction, then increased by 1.4% (–0.03, 3.1; *P* = 0.15) following resumption of habitual activity to a value similar to that observed at baseline, the overall change being 0.4% (–1.8, 2.6; *P* = 1.00). All vascular function data is summarized in Table 2. There was no significant overall effect of physical activity on baseline (*P* = 0.07) or peak diameter (*P* = 0.07). Upon resumption of habitual activity, structural properties of the artery were comparable to baseline [baseline 0.01 mm (0.00, 0.02); *P* = 0.49; peak 0.01 (0.00, 0.01); *P* = 0.42]. There was no effect of physical inactivity on sheer rate AUC (*P* = 0.86) or time to peak dilation (*P* = 0.42).

**TABLE 2:** Data of the brachial artery vascular function at baseline, following 14 days of physical inactivity and 14 days resumption to habitual activity.

	Baseline	Step-reduction	Resuming activity	*P*	*d*
Flow-mediated dilation; FMD (%)	8.1 (6.8, 9.3)	6.2 (5.2, 7.3)	7.6 (6.5, 8.8)	0.04	0.117
Baseline diameter (mm)	0.36 (0.34, 0.39)	0.34 (0.32, 0.37)	0.36 (0.33, 0.38)	0.07	0.102
Peak diameter (mm)	0.39 (0.36, 0.42)	0.37 (0.35, 0.40)	0.39 (0.36, 0.41)	0.07	0.106
Shear rate AUC (s^–1^ × 10^3^)	25331 (22862, 32089)	26954 (22862, 31047)	26478 (20757, 32199)	0.86	0.005
Time to peak (s)	58.5 (47.2, 69.8)	64.1 (56.1, 72.2)	65.3 (56.4, 47.1)	0.41	0.031

### Cardiorespiratory Fitness

$\dot{\text{V}}\,{\text{O}}_{2}$ peak relative to body mass decreased following step-reduction by 2.6 kg/ml/min (0.5, 4.8; *P* = 0.012; Figure 2B). Significant outcomes were also found for absolute $\dot{\text{V}}\,{\text{O}}_{2}$ which decreased by 0.2 L/min (0.1, 0.3; *P* = 0.013) and $\dot{\text{V}}\,{\text{O}}_{2}$ peak relative to lean body mass which decreased by 3.5 kg/ml/min (0.3, 6.7; *P* = 0.029).

### Lean Body Mass

Following step-reduction, lower limb lean mass decreased by 0.2 kg (0.2, 0.4*; P* = 0.029; Figure 2C), while upper limb lean mass and total body lean mass did not significantly change (*P* > 0.05).

### Liver and Body Fat

Following step-reduction, IHCL (liver fat) increased by 0.2% (0.1, 0.5; *P* = 0.022; Figure 2E), total body fat increased by 0.5% (0.1, 0.9; *P* = 0.014; Figure 2D) and android fat increased by 0.9% (0.1, 1.6; *P* = 0.017). However, changes in gynoid fat fell short of statistical significance (*P* = 0.078) and no significant difference was observed in IMCL (skeletal muscle lipid) (*P* = 0.199).

### Metabolic Markers

Following 14 days of step-reduction whole-body IS (Matsuda index) had decreased by 0.6 (0.1, 1.2; *P* = 0.020; Figure 2F). Circulating triglycerides increased by 0.2 mmol/L (0.1, 0.3; *P* = 0.016) following step-reduction. Total cholesterol, HDL, LDL, and HOMA-IR were not significantly altered by the intervention (*P* > 0.05).

### Return to Normal Activity

All clinical, biochemical, body composition cardiorespiratory fitness, and cardiovascular measures returned to levels comparable with baseline after 14 days resumption of habitual physical activity.

## Discussion

The results confirm our hypothesis that endothelial function becomes compromised (i.e., FMD is reduced) with a short-term reduction in physical activity and a concomitant increase in sedentary behavior. In parallel, step-reduction caused a decline in cardiorespiratory fitness and insulin sensitivity and promoted fat deposition (increased total body fat, waist circumference, and liver fat). Importantly, following resumption of normal activity, all changes were restored to baseline, in agreement with our previous metabolic findings during the same intervention (Bowden Davies et al., 2018). The observed changes in FMD are striking; a reduction of 1.8%, statistically controlling for baseline artery diameter, following step-reduction can be put into context by considering meta-analysis data: a 1% increase in FMD is associated with a relative risk reduction in CVD of 0.87 (Inaba et al., 2010). FMD returned to normal on resumption of activity. These data highlight the profound modulation physical activity may have on endothelial function, with potentially prognostic relevance for incident CVD.

**FIGURE 3:**
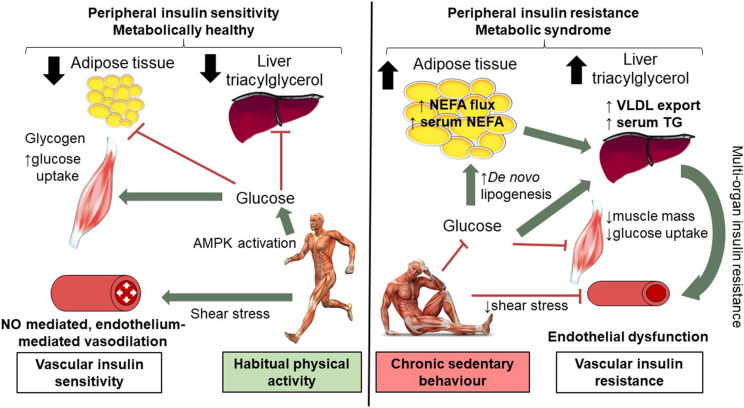
Mechanisms of short-term (14 days) physical inactivity and its role in the development of cardio-metabolic complications. Low levels of physical activity are associated with reduced shear stress; brachial artery endothelial function is compromised as a consequence of 14 days inactivity, as such NO mediated endothelium vasodilation is worsened. Low levels of physical activity alter insulin and AMPK signaling pathways in skeletal muscle and reduce GLUT4 translocation and glucose uptake. Skeletal muscle insulin resistance means additional glucose substrate for *de novo* lipogenesis in adipose tissue and the liver with expansion of adipose tissue and intra-hepatic lipid. Systemic insulin resistance, higher levels of circulating NEFA and TG are observed. Vascular insulin resistance may also contribute to peripheral insulin resistance in part due to reduced blood flow. In contrast, in habitually active individuals shear stress mediates endothelial-mediated vasodilatation (which together with intact skeletal muscle AMPK and insulin signaling) contributes to preserved peripheral insulin resistance. This figure is adapted from Bowden Davies et al. (2019) with full permissions from the authors and publisher.

Step-reduction research is in its infancy, although it already offers much mechanistic insight into the associated metabolic and musculoskeletal effects (recently reviewed Bowden Davies et al., 2019). The decline in cardiorespiratory fitness in this study confirms whole-body cardiovascular decompensation associated with inactivity, which may relate to the decompensation in endothelial function. The results from a variety of studies examining changes in vascular function with different durations and ‘doses’ of step reduction have yielded contrasting findings. An acute bout (4 h) of sedentary behavior has been shown to reduce superficial femoral artery blood flow, but not FMD (Carter et al., 2019). Whereas, FMD is worsened in other studies of 3 h to 5 days of reduced physical activity (Boyle et al., 2013; Restaino et al., 2016). Countermeasures have also been examined; local heating, can abolish the impairment of endothelial function associated with an acute episode of sedentary behavior (Restaino et al., 2016) or with reduced daily steps (Teixeira et al., 2017) by attenuating the reduction in limb blood-flow, and thus also in shear stress. Our study is the first to investigate endothelial function with a longer-term intervention with inactivity (∼2,000 steps/day). The present data demonstrates the ‘downstream’ vascular effects of alterations in physical activity; we speculate mechanisms may include a reduction in shear stress, a down regulation of nitric oxide (NO) bioavailability and likely minor structural vascular alterations (Kim et al., 2006). A proposed schematic is shown in Figure 3.

As a result of physical inactivity, we observed small but significant changes in metabolic profiles and body composition that would predispose individuals to the development of metabolic syndrome and/or type 2 diabetes: a decrease in insulin sensitivity, increase in plasma triglycerides (0.2 mmol/L), loss of (lower limb) lean body mass and deposition of total body and liver fat (0.2%). In terms of the twin cycle hypothesis (Taylor, 2008), it has been suggested that insulin sensitivity and liver fat mediate the association of physical activity with glycemic control (Koivula et al., 2020). Our previous study showed that changes in insulin sensitivity were specific to skeletal muscle (Bowden Davies et al., 2018). It therefore seems plausible the primary effect of reduced physical activity is peripheral insulin resistance, with secondary consequences in the liver including attenuated suppression of hepatic gluconeogenesis, and over-production of lipoproteins. Our intervention did not induce a significant change in IMCL (i.e., skeletal muscle lipid). However, recent findings employing unilateral limb suspension as a model of inactivity, caused a decrease in mitochondrial oxidative capacity accompanied by increased IMCL purporting a role for lipotoxicity-induced mitochondrial dysfunction in the development of insulin resistance (Bilet et al., 2020).

The worsened metabolic profile from reduced physical activity has also been associated with increased CVD risk and early atherosclerosis (Gastaldelli et al., 2009). Furthermore, non-alcoholic fatty liver disease (NAFLD) is linked with the pathogenesis of atherosclerosis (Brouwers et al., 2019). Elevated circulating triglycerides, reported to be causal in the pathogenesis of coronary artery disease (Do et al., 2013), combined with endothelial dysfunction, trigger low-grade inflammation which can over time cause vulnerable plaque formation. Indeed, the vascular and metabolic effects demonstrated here may provide a mechanistic basis whereby physical inactivity can cause or at least accelerate disease progression.

Individuals at high risk of (or already suffering from) cardio-metabolic disease, and older adults, may be more susceptible to the deleterious effects of inactivity. Understanding the mechanistic basis of this pathophysiology may aid implementation of non-pharmacological interventions, as well as help identify novel therapeutic molecular targets as ‘exercise-mimetics.’ The degree of decompensation observed after periods of inactivity in individuals with poorer baseline metabolic/musculoskeletal health or lower baseline physically activity may indeed be less effective; further research is needed to address this possibility. Such studies of course raise serious ethical questions: while young adults return rapidly to baseline health following resumption of normal activity, recovery is more prolonged in older, pre-diabetic adults (McGlory et al., 2017). It might be suggested that the changes could be attributed to reduced total energy expenditure in the absence of any dietary alterations. However, as Taylor suggests (Taylor, 2008), the twin cycle mechanism operates with *chronic* positive energy balance; here we observe marked changes to physiology that are short-term effects of physical inactivity as opposed to positive energy balance.

In this first study to examine the endothelial response to short-term changes to physical activity we acknowledge some limitations. For example, its wider validity to other cohorts (e.g., older adults and those at risk of/with disease) and those with lower baseline level of physical activity (i.e., <10,000 steps/day). We acknowledge the absence of a control group: inclusion of an additional group of participants who persist with their habitual physical activity, having the same serial measurements, would have provided useful insight; a randomized cross-over design would be optimal. It may be that a longer duration of step-reduction might have revealed more effects. Key strengths of the study include complete objective monitoring of participants (confirming protocol adherence) with comprehensive and validated methods of metabolic and body composition assessment that provide a whole-body, integrative physiological assessment of the impact of reduced physical activity. Furthermore, this method of assessing endothelial function is considered the gold standard in the most recent expert consensus and evidence-based recommendations (Thijssen et al., 2019).

In summary, in young, non-obese adults, even short-term physical inactivity and increased sedentary behavior induces significant decline in endothelial function, a surrogate marker for incident CVD. In line with previous observations, a decline in cardiorespiratory fitness was accompanied by whole body insulin resistance and central and liver fat deposition. All changes were entirely reversible with resumption of normal levels of physical activity. Targeting sedentary behavior and promoting physical activity has clear implications to addressing the burden of type 2 diabetes and CVD.

## Data Availability Statement

The raw data supporting the conclusions of this article will be made available by the authors, without undue reservation.

## Ethics Statement

The studies involving human participants were reviewed and approved by North West – Liverpool Central Ethics Committee, Liverpool, England, United Kingdom. The patients/participants provided their written informed consent to participate in this study.

## Author Contributions

VS, DC, GK, and JW conceived the study, or parts of the study. KBD, VS, JN, and DC generated the data. VS analyzed the FMD data. GK analyzed the MRS data. KM analyzed the nutritional data. JCGH and JAH acquired the DXA data. KBD and AT statistically analyzed and interpreted the data. All the authors participated in preparation of the manuscript and approved the final version for publication. KBD and VS are the guarantors of this work.

## Conflict of Interest

The authors declare that the research was conducted in the absence of any commercial or financial relationships that could be construed as a potential conflict of interest.

## Footnotes

1 https://www.nutritics.com/p/home

## Supplementary Material

Supplementary Table 1 Dietary intake and composition at baseline, following 14 days of physical inactivity and 14 days resumption to habitual activity.
