# Supplementary material for: Short-Term Physical Inactivity Induces Endothelial Dysfunction
Source: Front Physiol. 2021 Apr 9;12:659834. doi: 10.3389/fphys.2021.659834 (PMC8064120; doi:10.3389/fphys.2021.659834)
Supplement: Supplementary Table 1 — Dietary intake and composition at baseline, following 14 days of physical inactivity and 14 days resumption to habitual activity. [file Table_1.docx]

|  | Baseline | Step-reduction | Resuming activity | *P* |
| --- | --- | --- | --- | --- |
| Energy consumption (KJ/day) | 8322 (7301, 9344) | 7926 (6959, 8892) | 8054 (7274, 8834) | 0.637 |
| Carbohydrate (g/day) | 217 (192, 243) | 206 (181, 230) | 214 (190, 239) | 0.671 |
| Protein (g/day) | 93 (76, 97) | 85 (74, 97) | 87 (78, 97) | 0.458 |
| Fat (g/day) | 77 (62, 91) | 77 (63, 90) | 74 (62, 85) | 0.808 |

**Supplementary Table 1** Dietary intake and composition at baseline, following 14 days of physical inactivity and 14 days resumption to habitual activity
